# Synergistic lethality in chronic myeloid leukemia – targeting oxidative phosphorylation and unfolded protein response effectively complements tyrosine kinase inhibitor treatment

**DOI:** 10.1186/s12885-023-11623-6

**Published:** 2023-11-27

**Authors:** Lukas Häselbarth, Sara Gamali, Domenica Saul, Manuela Krumbholz, Romy Böttcher-Loschinski, Martin Böttcher, Deyu Zou, Markus Metzler, Axel Karow, Dimitrios Mougiakakos

**Affiliations:** 1 Department of Pediatrics and Adolescent Medicine, University Hospital Erlangen, Erlangen, Germany; 2 Comprehensive Cancer Center Erlangen-European Metropolitan Area Nuremberg (CCC-ER-EMN), Erlangen, Germany; 3 Interdisciplinary Centre for Clinical Research (IZKF), Erlangen, Germany; 4 Department of Internal Medicine 5, Hematology and Oncology, University Hospital Erlangen, Erlangen, Germany; 5 Department of Hematology and Oncology, Medical Center, Otto-Von-Guericke University Magdeburg, Magdeburg, Germany; 6 Health Campus Immunology, Inflammation and Infectiology (GC-I3), Medical Center, Otto-Von-Guericke University Magdeburg, Magdeburg, Germany

**Keywords:** CML, TKI, UPR, Oxidative phosphorylation, Oligomycin, Thapsigargin, ATF4, 8-chloroadenosine, Olaparib, Caspase 3, PARP

## Abstract

Chronic myeloid leukemia (CML) is effectively treated with tyrosine kinase inhibitors (TKIs), targeting the BCR::ABL1 oncoprotein. Still, resistance to therapy, relapse after treatment discontinuation, and side effects remain significant issues of long-term TKI treatment. Preliminary studies have shown that targeting oxidative phosphorylation (oxPhos) and the unfolded protein response (UPR) are promising therapeutic approaches to complement CML treatment. Here, we tested the efficacy of different TKIs, combined with the ATP synthase inhibitor oligomycin and the ER stress inducer thapsigargin in the CML cell lines K562, BV173, and KU812 and found a significant increase in cell death. Both, oligomycin and thapsigargin, triggered the upregulation of the UPR proteins ATF4 and CHOP, which was inhibited by imatinib. We observed comparable effects on cell death when combining TKIs with the ATP synthase inhibitor 8-chloroadenosine (8-Cl-Ado) as a potentially clinically applicable therapeutic agent. Stress-related apoptosis was triggered via a caspase cascade including the cleavage of caspase 3 and the inactivation of poly ADP ribose polymerase 1 (PARP1). The inhibition of PARP by olaparib also increased CML death in combination with TKIs. Our findings suggest a rationale for combining TKIs with 8-Cl-Ado or olaparib for future clinical studies in CML.

**Supplementary Information:**

The online version contains supplementary material available at 10.1186/s12885-023-11623-6.

## Background

Chronic myeloid leukemia (CML) is a myeloproliferative disorder that is effectively treated with tyrosine kinase inhibitors (TKIs), targeting the BCR::ABL1 fusion protein. However, a significant amount of patients show resistance to therapy or experience substantial TKI side effects. Additionally, only a proportion of patients can successfully discontinue TKI medication and reach sustainable treatment-free remission (TFR). Therefore, complementing therapeutic options, aiming at a more effective and rapid achievement of deep and sustained molecular response, are needed. This would enable timely termination of TKI therapy, minimizing their long-term adverse effects like cardiovascular and pulmonary toxicities or growth retardation in children [1–4].

Additional targeting of cancer cell metabolism represents a promising approach and has already been shown to enhance the treatment efficiency in various malignancies [5]. In CML, Kuntz et al. showed that targeting oxidative phosphorylation (oxPhos) is a valuable tool to eradicate therapy-resistant leukemia stem cells (LSCs) [6], suggesting a strong dependency of CML cell survival on the activity of the respiratory chain. Accordingly, it was observed that the mitochondrial ATP synthase inhibitor oligomycin A rendered leukemia cells more sensitive to TKI treatment [7]. Moreover, glycolysis, as another important ATP producing signaling pathway, was shown to be inhibited by imatinib [8].

Here, we elucidated how the TKIs imatinib, dasatinib, nilotinib, and asciminib affect mitochondrial respiration and glycolysis of the *BCR::ABL1*-positive cell lines K562, BV173, and KU812 and the *BCR::ABL1*-negative control cell line HL60. Additionally, we examined synergistic effects of imatinib and different oxPhos-inhibitors, including oligomycin, on CML cell survival. Yong et al. showed that ATP from the mitochondria is transported to the endoplasmic reticulum (ER) and that a sarcoplasmic/endoplasmic reticulum calcium ATPase (SERCA) gradient across the ER membrane is needed for the import of ATP into the ER [9]. In the ER, ATP can be used for the unfolded protein response (UPR). Therefore, we hypothesized that the UPR, as an important modulator of apoptosis and potentially vulnerable target in CML cells, might be triggered by oxPhos-inhibition. In particular, we tested whether oligomycin and thapsigargin, an inhibitor of the SERCA gradient across the ER membrane, affect the expression of different UPR proteins, including the activating transcription factor 4 (ATF4) and the C/EBP (CCAAT-enhancer-binding protein) homologous protein (CHOP). Moreover, we assumed that the combination of TKIs and oligomycin or thapsigargin might further reduce the viability of CML cells via modulation of UPR expression. To complement our mechanistic findings with oligomycin and thapsigargin, we additionally tested the potential therapeutic candidate 8-chloroadenosine (8-Cl-Ado). This agent is currently investigated in patients with acute myeloid leukemia (AML) in clinical studies (NCT02509546, NCT05263284) and has, similar to oligomycin, shown to inhibit oxygen consumption rate (OCR) and ATP synthase [10, 11]. We analyzed the effects of TKIs, combined with 8-Cl-Ado, on CML cell death to elucidate potential benefits for future CML treatment and examined, which apoptosis signaling pathways are triggered by oligomycin, thapsigargin, and 8-Cl-Ado in combination with imatinib. Having identified changes in the activity of the central apoptotic mediator caspase 3, which is a downstream target of UPR signaling and inhibitor of poly ADP ribose polymerase (PARP), we subsequently also analyzed the cleavage of PARP and tested whether the therapeutic PARP-inhibitor olaparib affects CML cell death in combination with TKIs.

## Methods

### Cell culture

All cell lines were purchased commercially from the German Collection of Microorganisms and Cell Cultures GmbH (DSMZ, Brunswick, Germany). K562, BV173, and KU812 are *BCR::ABL1*-positive human blast phase (BP) CML cell lines and the HL60 cell line is a human AML cell line, which we used as a *BCR::ABL1*-negative control. Cells were cultured at a density of 2.5–5 × 10^5^/ml in RPMI1640, supplemented with 10% (K562, HL60) or 20% (BV173, KU812) fetal calf serum (FCS, ThermoFisher Scientific, Waltham, USA), 2 mM L-Glutamine (Sigma-Aldrich, St. Louis, USA), and 40 U/ml Penicillin/Streptomycin (P/S, ThermoFisher Scientific). Inhibitor concentrations (Suppl. Table 1) were set at a limit that they did not induce > 20% in vitro specific cell death (SCD), compared to the untreated controls. Cells were treated with/without inhibitors for 24 h at 37 °C and 5% CO_2_. When cells were co-treated with combinations of two inhibitors, they were added simultaneously, if not otherwise stated in the figure captions.

### Analysis of viability, protein expression, and metabolic fitness by FACS

Cell lines were examined using a FACS (Fluorescence-activated cell sorting) Canto II flow cytometer (BD Biosciences, New Jersey, USA) and subsequently analyzed with the FlowJo software Version 10.6.2 (TreeStar, Ashland, USA). 10.000 cells/sample were manually gated for single living cells. For viability measurements also dead cells were included in the analysis. The toxicity of inhibitors was measured via 7-Aminoactinomycin (7AAD, Biolegend, San Diego, USA). 7AAD was incubated for 10–15 min at RT. Cells negative for 7AAD were characterized as vital. In some analyses, stainings with Zombi Aqua™ (Biolegend) were included for the distinction of dead and living cells. Therefore, washing steps were performed with phosphate buffered saline (PBS, ThermoFisher Scientific) For the staining of surface markers (αCD36 (cluster of differentiation 36), αCD98, αGlut-1 (glucose transporter 1)), washing steps were carried out using FACS buffer, consisting of PBS, supplemented with 0.25% bovine serum albumin (Carl Roth, Karlsruhe, Germany). Antibodies against surface proteins were incubated for 20 min at 4 °C. For intracellular staining of cleaved (Cl) PARP (Cl Asp214), cells were fixed and permeabilized with the BD Cytofix/Cytoperm™ Kit (BD Biosciences). Antibodies against PARP1 were incubated for 30 min at 4 °C.

The glucose- and fatty acid (FA) uptake was analyzed by 6-NBDG (ThermoFisher Scientific) and Bodipy™ FL C_16_ (ThermoFisher Scientific) as previously described [12].

For the analysis of mitochondrial fitness, cells were stained with TMRE (tetramethylrhodamine, ethyl ester), MitoTracker™ Green FM, MitoSOX™ Red Mitochondrial Superoxide Indicator, and CellROX™ Deep Red (all ThermoFisher Scientific). These stainings were performed for the analysis of mitochondrial membrane potential (ΔΨm), mass, and generation of mitochondrial and cellular reactive oxygen species (ROS), respectively.

All antibodies and reagents for flow cytometry are shown in Supplementary Table 2. Exemplary gating strategies for the FACS analyses are shown in Supplementary Fig. 1.

### RNA expression of target genes

RNA of cell lines was isolated with the innuPREP DNA/RNA Mini Kit (Analytik Jena GmbH, Jena, Germany) according to the manufacturer’s instructions. cDNA was generated, using the Superscript™ First-Strand Synthesis System (ThermoFisher Scientific) and the Mastercycler® Nexus (Eppendorf, Hamburg, Germany). mRNA expression was quantified by quantitative polymerase chain reaction (qPCR), using the Quantitect SYBR Green PCR Kit (Qiagen, Hilden, Germany) and a Rotor-Gene Q device (Qiagen). The experiments were carried out with two technical replicates. The relative expression of target genes was calculated by the 2^−∆∆CT^ technique, using Beta-actin as a housekeeping gene. All primers are shown in Supplementary Table 3.

### Analysis of the metabolic phenotype and ATP production rate

The metabolic phenotype was determined by metabolic flux analyses with an XF96e Extracellular Flux Analyzer (Agilent Technologies, Santa Clara, USA) as previously described [12, 13]. This device measures the cellular OCR (pmol/min) and extracellular acidification rate (ECAR in mpH/min), which are indicative of oxPhos activity and glycolysis, respectively. For measurement of ATP, produced by oxPhos and glycolysis (= mitochondrial and glycolytic ATP production rate; mitoATP and glycoATP in pmol ATP/min) the Seahorse XF Real-Time ATP Rate Assay Kit (Agilent Technologies) was used. In short, OCR- and ECAR-values were measured before and after the serial injection of first, the mitochondrial complex V inhibitor oligomycin, and second, the complex I/III inhibitors rotenone (Rot) and antimycin A (AA). Subsequently, mitoATP and glycoATP were calculated according to the manufacturer’s instructions [14]. The total ATP production rate is defined as the sum of mitoATP and glycoATP. Data were acquired by the WAVE software version 2.6.1 (Agilent Technologies).

### Analysis of protein expression by western blot

Cells were lysed with radioimmunoprecipitation assay (RIPA) buffer (Sigma-Aldrich) and the protein concentration was determined via bicinchoninic acid (BCA) assay (ThermoFisher Scientific). Protein extracts were fractioned by sodium dodecyl sulfate polyacrylamide gel electrophoresis (SDS-PAGE) and transferred to a polyvinylidene fluoride (PVDF) membrane (ThermoFisher Scientific). Primary antibodies were incubated overnight at 4 °C and horseradish peroxidase (HRP)-conjugated secondary antibodies for 1 h at RT or 4-6 h at 4 °C (Suppl. Table 4). Protein bands were detected with chemiluminescent substrate (ThermoFisher Scientific and Cell Signaling Technology, Danvers, USA) at an Amersham Imager 600 (GE HealthCare, Chicago, USA). For quantification of the protein bands, the mean grey value of the protein of interest was measured with ImageJ (National Institute of Health, Bethesda, USA) and normalized by the mean grey value of a housekeeper (Beta-actin or GAPDH).

### Lactate measurement

Measurements of lactate content (mg/dl) in cell supernatants were performed with the Super GL compact (Hitado GmbH, Möhnesee, Germany) according to the manufacturer’s instructions.

### Measurement of caspase 3 activity

Changes in procaspase 3 and Cl caspase 3 were identified by a membrane-based sandwich immunoassay (Proteome Profiler Human Apoptosis Array Kit, RnD Systems, Minneapolis, USA). Protein expression was measured with a chemiluminescent reagent at the Amersham Imager 600. Protein circles on the array membrane were quantified by measuring the pixel density with ImageJ. Caspase 3-activity was further determined with the semi-quantitative Caspase-3, Caspase-8, and Caspase-9 Multiplex Activity Assay Kit (fluorometric, Abcam, Cambridge, UK) according to the manufacturer’s instructions. Fluorescence was measured at a Spectra Max M3 device (Molecular Devices, San José, USA) using the software SoftMax Pro 6.4 (Molecular Devices).

### Statistical analysis

**Fig. 1:**
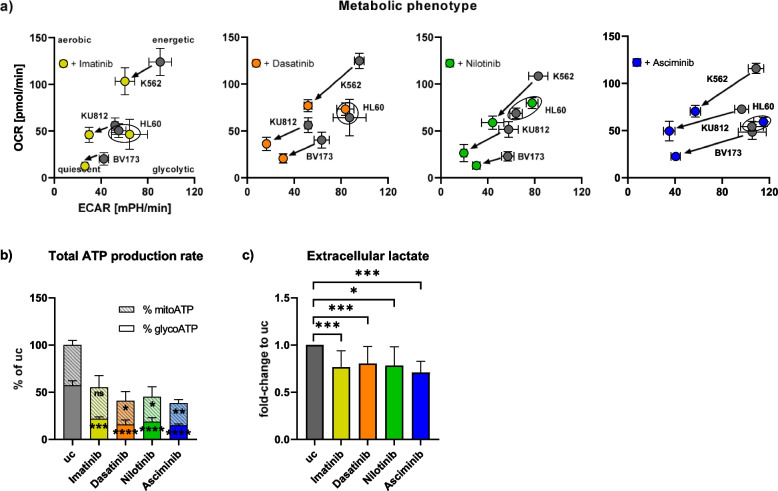
TKIs affect ATP synthesis of CML cells. The human CML cell lines K562, BV173, and KU812 were treated with/without the TKIs imatinib (yellow), dasatinib (orange), nilotinib (green), or asciminib (blue) for 24 h. **a** The metabolic phenotype was elucidated by metabolic flux analysis, measuring OCR and ECAR. The HL60 cell line was used as a BCR::ABL1-negative control. **b** ATP production rate was observed by Real-Time ATP Rate Assay. Data from the three CML cell lines were pooled. Comparison of % mitoATP and % glycoATP of the TKI-treated groups with the uc, respectively (*n* = 3). **c** The lactate content of cell supernatants was measured with the Super GL compact. Data from the three CML cell lines were pooled. Comparison of the TKI-treated groups with the uc (n = 9–18). ECAR = extracellular acidification rate, glycoATP = glycolytic ATP production rate, mitoATP = mitochondrial ATP production rate, OCR = oxygen consumption rate, TKI = tyrosine kinase inhibitor, uc = untreated control. Data are represented by the mean and standard deviation of the biological replicates. **** *p* < 0.0001 *** *p* < 0.001, ** *p* < 0.01, * *p* < 0.05, ns = not significant

**Fig. 2:**
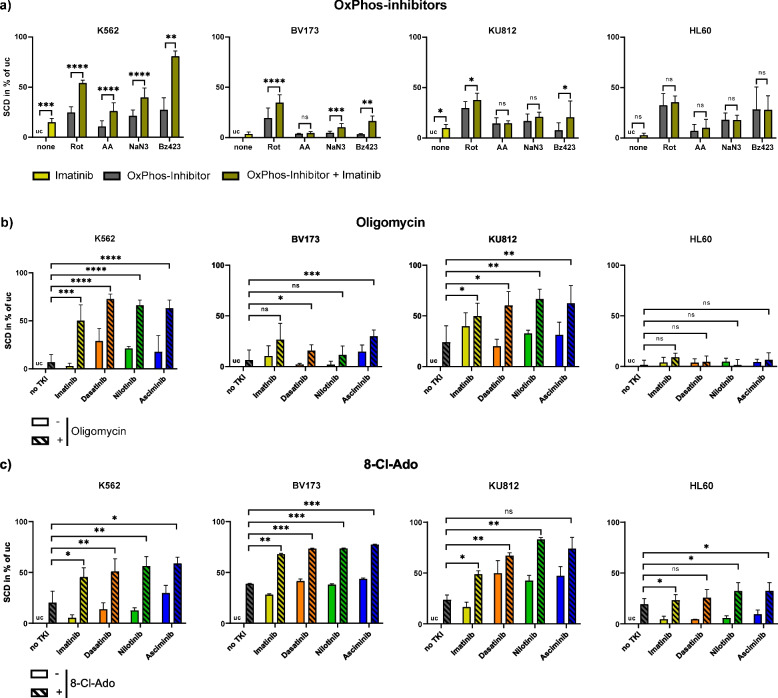
OxPhos-inhibitors and 8-Cl-Ado increase the lethality of CML cells in combination with TKIs. The human CML cell lines K562, BV173, and KU812 and the BCR::ABL1-negative control cell line HL60 were treated with TKIs for 24 h in combination with different mitochondrial inhibitors. The cells were stained with 7AAD and cell death was measured by FACS. **a** Inhibitors of complex I (Rot), III (AA), IV (NaN_3_), and V (Bz423) of the mitochondrial respiratory chain, combined with imatinib. Comparison of oxPhos-inhibitor + imatinib with oxPhos-inhibitor only (*n* = 4 per cell line), **b** Complex V inhibitor oligomycin, combined with imatinib (yellow), dasatinib (orange), nilotinib (green), or asciminib (blue). Comparison of oligomycin + TKI with oligomycin only each (*n* = 3–4 per cell line), **c** OxPhos-inhibitor 8-Cl-Ado in combination with imatinib, dasatinib, nilotinib, or asciminib. Comparison of 8-Cl-Ado + TKI with 8-Cl-Ado only each (*n* = 3 per cell line). All data show the SCD in % of the uc (= 0%) and are represented by the mean and standard deviation of the biological replicates. 8-Cl-Ado = 8-chloroadenosine, AA = antimycin A, OxPhos = oxidative phosphorylation, Rot = rotenone, SCD = specific cell death, TKI = tyrosine kinase inhibitor, uc = untreated control. **** *p* < 0.0001, *** *p* < 0.001, ** *p* < 0.01, * *p* < 0.05, ns = not significant

Data analysis was performed using the GraphPad Prism Version 9 software (GraphPad Software, San Diego, USA). The figure captions describe, which treatment groups were statistically compared to each other. Expression of metabolic parameters (Suppl. Figure 2) and oxPhos complex expression (Suppl. Figure 4) were analyzed by unpaired two-tailed t-tests with Welch’s correction. MitoATP, glycoATP (Fig. 1b), and toxicity of oxPhos-inhibitors (Fig. 2a) were analyzed via RM two-way ANOVA, followed by Šidák’s multiple comparison test. For all other statistical comparisons, the RM one-way ANOVA or Mixed-effects analysis with Geisser-Greenhouse correction was performed, dependent on the existence of missing data points or excluded outliers, followed by Dunnett’s multiple comparison test, respectively. Statistical outliers were identified by the ROUT (robust regression and outlier removal) method (Q = 1%).

## Results

### TKIs inhibit ATP synthesis of CML cells by glycolysis- and oxPhos-inhibition

We treated *BCR::ABL1*-positive human CML cell lines (K562, BV173, and KU812) with imatinib, dasatinib, nilotinib, and asciminib. Subsequently, we assessed > 20 metabolic parameters by FACS or qPCR. Overall, the bioenergetic state of TKI-treated CML cells was found to be altered with changes in glucose- and FA metabolism (Suppl. Figure 2). These observations were further supported by dynamic metabolic flux analyses revealing a clear TKI-triggered attenuation of ECAR, reflecting reduced aerobic glycolysis, and a slight reduction of OCR, indicative of oxPhos (Fig. 1a). Although K562, BV173, and KU812 cells had a different basal metabolic phenotype, the relative shift to a more quiescent metabolic phenotype by the TKIs was comparable in all CML cell lines. In contrast, there were no TKI-induced effects present in the *BCR::ABL1*-negative cell line HL60. Glycolysis inhibition was also shown by a significant TKI-induced reduction of glycoATP and decreased lactate levels in the supernatant of *BCR::ABL1*-positive CML cell lines (Fig. 1b,c). The unpooled data (Suppl. Figure 3a,b) show that this effect occurred similarly in all CML cell lines.

This is in line with the reduction of RNA expression of enzymes, involved in glycolysis, by TKIs (i.e. *eno1*, *ldha*, Suppl. Figure 2). MitoATP of CML cells was also significantly reduced by dasatinib, nilotinib, and asciminib while the decrease was not significant for imatinib (Fig. 1b). Generally, TKI-induced mitoATP-reduction was lower than glycoATP-reduction (Fig. 1b) and slightly more pronounced in K562- and BV173 than in KU812 (Suppl. Figure 3a). We observed no reduction of oxPhos complex protein expression by imatinib (Suppl. Figure 4), independent of the cell line.

### OxPhos-inhibition and ER stress induction increase SCD in combination with TKIs

Focusing on the synergistic effects of TKIs and oxPhos targeting compounds, we treated K562, BV173, KU812, and HL60 cells with inhibitors of complex I, III, IV, and V of the mitochondrial respiratory chain. For all CML cell lines, we found an increased lethality when treating cells simultaneously with imatinib and the complex I inhibitor Rot or the complex V inhibitor Bz423 in comparison to the samples with oxPhos-inhibitors only. This synergistic effect was most pronounced in K562 cells (Rot: From 24% (without imatinib) to 54% (with imatinib), Bz423: From 27% (without imatinib) to 81% (with imatinib)), which also showed, in contrast to KU812, an increase of cell death when treated with combinations of imatinib and AA or NaN_3_ (Fig. 2a). For HL60 cells, we observed no combinatory toxic effects of oxPhos-inhibitors and imatinib in comparison to the samples with oxPhos-inhibitors only. Also, the ER stress inducer thapsigargin increased the SCD of the CML cells in combination with imatinib, significantly in K562 cells (from 10 to 32%) and non-significantly in BV173 cells (from 2 to 17%) and KU812 cells (from 26 to 68%) compared to thapsigargin only, while there was no combinatory effect on HL60 cells (Suppl. Figure 5). Additionally, we observed an increased lethality through the combination of the complex V inhibitor oligomycin, and the TKIs imatinib, dasatinib, nilotinib, and asciminib in all CML cell lines (Fig. 2b). Oligomycin alone and in combination with TKIs was non-toxic for HL60 cells. In a potentially therapeutic approach, we tested the mitochondrial complex V inhibitor 8-Cl-Ado. In all CML cells, TKIs increased the SCD when combined with 8-Cl-Ado (from 20–39% (without TKI) to 45–68% (with imatinib), 51–73% (with dasatinib), 56–83% (with nilotinib), and 59–77% (with asciminib), Fig. 2c). A slight increase of SCD by 8-Cl-Ado was also detected in HL60 cells but the overall toxicity in combination with the TKIs was lower as compared to the *BCR::ABL1*-positive CML cell lines (increase of 19% to 23–32%).

### Oligomycin triggers UPR upregulation similar to thapsigargin, which is inhibited by imatinib

**Fig. 3:**
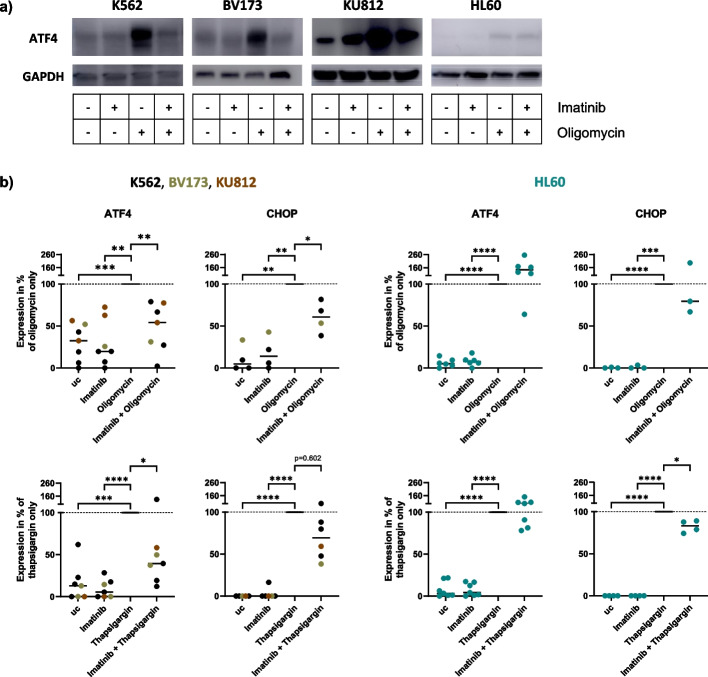
Oligomycin induces ER stress similar to thapsigargin, and imatinib blocks UPR upregulation. The human CML cell lines K562, BV173, and KU812 and the BCR::ABL1-negative control cell line HL60 were treated with/without imatinib and oligomycin for 24 h. Thapsigargin was added in the last 4 h of treatment. Protein expression was observed by western blot. **a** Images representatively show the ATF4 expression of all cell lines, treated with different combinations of imatinib and oligomycin with GAPDH as a reference protein. Uncropped blots are presented in Supplementary Fig. 6. **b** Western blot quantifications of ATF4 and CHOP (mean grey value, normalized to GAPDH or Beta-actin). Expression of ATF4 and CHOP was normalized to the samples with oligomycin only (top) or thapsigargin only (bottom) by setting these samples to an expression of 100%. For the statistical analysis, the ATF4-/CHOP-expression of oligomycin only or thapsigargin only, respectively, were compared to the other treatment groups. uc = untreated control. Data are represented by the mean gray value. **** *p* < 0.0001, *** *p* < 0.001, ** *p* < 0.01, * *p* < 0.05

Since oxPhos in the mitochondria and the UPR in the ER are connected processes through the dependency of UPR on mitochondrial ATP, we investigated whether oligomycin affects the UPR expression in the ER, similar to thapsigargin. Figure 3a shows representative western blots of the UPR protein ATF4 for the CML cell lines K562, BV173, and KU812, and the *BCR::ABL1*-negative cell line HL60, treated with/without imatinib and oligomycin. There was an upregulation of ATF4 by oligomycin in all cell lines, compared to the untreated controls and imatinib-treated samples (relative induction, Fig. 3b) but the HL60 cell line had a trend to a less absolute ATF4 induction by oligomycin (exemplarily shown in Fig. 3a). The oligomycin-induced ATF4 upregulation in the CML cell lines was decreased by 52% when cells were additionally treated with imatinib while ATF4 expression roughly remained unchanged in HL60 cells (Fig. 3b). Analogous effects were observed at thapsigargin-treated CML cells and for the UPR protein CHOP (Fig. 3b).

### Combining imatinib with oligomycin, thapsigargin, or 8-Cl-Ado triggers cleavage of caspase 3

**Fig. 4:**
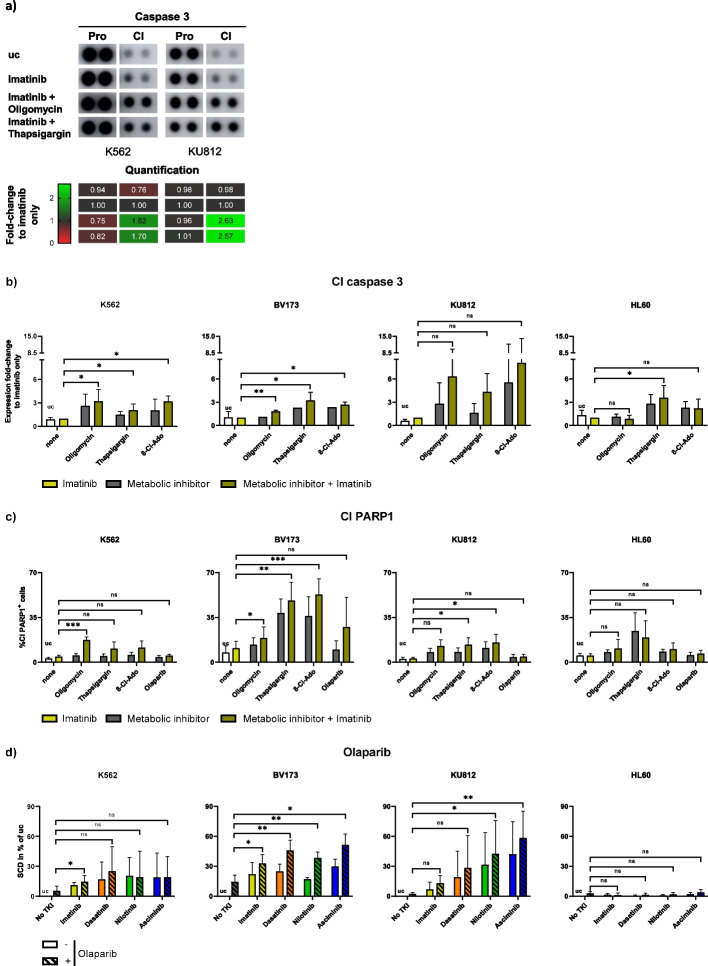
Combination of imatinib with metabolic inhibitors triggers apoptosis via caspases and cleavage of PARP1. **a** The human CML cell lines K562 and KU812 were treated with/without imatinib and oligomycin or thapsigargin for 24 h. Subsequently, the expression of apoptosis-related proteins (shown: Procaspase 3 and Cl caspase 3) was determined via a membrane-based sandwich immunoassay (each cell line *n* = 1). Full-length blots are presented in Supplementary Fig. 7. **b** K562, BV173*,* KU812, and the *BCR::ABL1*-negative control cell line HL60 were treated with/without imatinib and oligomycin, thapsigargin*,* or 8-Cl-Ado for 24 h. Subsequently, protein expression of Cl caspase 3 was determined via a caspase multiplex activity assay kit. Data are represented by the mean expression of Cl caspase 3 and SD. Comparison of groups with metabolic inhibitors + imatinib with imatinib only each (*n* = 4–6 per cell line). **c** K562, BV173*,* KU812, and HL60 cells were treated with/without imatinib in combination with/without oligomycin, thapsigargin, 8-Cl-Ado, or olaparib for 24 h and stained for Cl PARP1. Data are represented by the mean % of PARP1^+^ cells and SD. Comparison of groups with metabolic inhibitors + imatinib with imatinib only each (*n* = 4–6 per cell line). **d** K562, BV173*,* KU812, and HL60 cells were treated with/without imatinib, dasatinib, nilotinib, or asciminib in combination with/without the PARP-inhibitor olaparib for 24 h, stained with 7AAD*,* and cell death was measured via FACS. Data are represented by the mean SCD in % of the uc and SD. Comparison of groups with olaparib + TKIs with olaparib only each (*n* = 3–4 per cell line). 8-Cl-Ado = 8-chloroadenosine, Cl = cleaved, PARP = poly ADP ribose polymerase, SCD = specific cell death, SD = standard deviation, uc = untreated control. *** *p* < 0.001, ** *p* < 0.01, * *p* < 0.05, ns = not significant

Analyzing which apoptosis pathways downstream to UPR signaling were activated when combining imatinib with oligomycin or thapsigargin, we carried out membrane-based sandwich immunoassays with the cell lines K562 and KU812 (Fig. 4a). We found a strong increase in Cl caspase 3 when combining imatinib with oligomycin or thapsigargin compared to the control with imatinib only, while the expression of procaspase 3 was barely affected. The uncropped array blots are shown in Supplementary Fig. 7. A caspase multiplex activity assay confirmed the increase in Cl caspase 3 for K562, KU812, and also BV173 and additionally, for the combination imatinib + 8-Cl-Ado (Fig. 4b). At KU812, the effects were the strongest but not significant due to high variations of the biological replicates. The data of Fig. 4b also indicate that the combinatory increase of caspase 3 cleavage was mainly caused by oligomycin, thapsigargin, and 8-Cl-Ado, while imatinib only had a small effect. In the HL60 cell line, a caspase 3-induction was observed for imatinib + thapsigargin in comparison with imatinib only, whereas oligomycin had no and 8-Cl-Ado a slight non-significant effect.

### Combined treatment of TKIs and the PARP-inhibitor olaparib increase cell death of CML cells

Since metabolic inhibitors had positive effects on the activity of caspase 3, we tested whether this affects the cleavage of the downstream target PARP1 via FACS. In all CML cell lines, we observed a strong induction of PARP1 cleavage when combining imatinib with oligomycin, thapsigargin, or 8-Cl-Ado, compared to imatinib only (Fig. 4c). The treatments with single agents suggest that PARP1 cleavage was mainly caused by oligomycin, thapsigargin, and 8-Cl-Ado and to a smaller extent by imatinib, which is in line with the caspase 3 data (Fig. 4b). The PARP-inhibitor olaparib had no effect on the cleavage of PARP1 in combination with imatinib (Fig. 4c), but showed a combinatory toxic effect on BV173 and KU812 cells, when combined with imatinib, dasatinib, nilotinib, or asciminib (Fig. 4d). In K562 cells, combinatory toxic effects were lower or non-existent. In HL60 cells, we observed slight inductions of PARP1 cleavage which were not significant (Fig. 4c). Additionally, olaparib had no toxic effects on HL60 cells (Fig. 4d).

## Discussion

The examination of tumor metabolism has opened up new therapeutic intervention strategies for different malignant diseases, including leukemia. In CML, complementing the conventional TKI monotherapy with agents, targeting the ATP production of cancer cells, is a promising approach. A preliminary study showed that TKI-resistant stem cells were vulnerable to imatinib in combination with tigecycline, an inhibitor of mitochondrial protein translation [6]. In addition, the oxPhos-inhibitor oligomycin has been shown to sensitize leukemia cells to TKI treatment [7]. In our study, based on three different CML cell lines, we show, that especially dasatinib, nilotinib, and asciminib were effective in reducing mitoATP. Furthermore, glycolysis, an alternative way of ATP production, was strongly attenuated by TKIs, which is in accordance with previous data with imatinib [8]. Additionally, glycolysis attenuation by the hexokinase II inhibitor 2-deoxy-d-glucose (2-DG) reduced the viability of CML cells [15]. The dependency of CML cells on glycolysis is also in line with the findings of Barger et al., who observed glycolysis activation and glucose-dependent survival by BCR::ABL1 [16]. Here, we discovered synergistic effects of imatinib and different oxPhos-inhibitors on CML cell death and apoptosis pathways, which are triggered in comparison to imatinib only in order to detect metabolic vulnerabilities of CML cells that might be used for new therapeutic approaches. In all three CML cell lines, we found vulnerabilities for the oxPhos complex inhibitors Rot, Bz423, and oligomycin in combination with imatinib, suggesting that CML cells are sensitive to complex I- or V-inhibition.

In addition, we observed in K562, BV173, and KU812 cell lines that oligomycin, similar to the Ca^2+^-dependent SERCA-inhibitor thapsigargin, triggered the upregulation of the transcription factors ATF4 and CHOP that are involved in the UPR of the ER. Yong et al. showed that mitochondria supply ATP to the ER, which is transported into the ER via SLC35B1/AXER (Solute carrier family 35 member B1/ ATP/ADP exchanger in the ER membrane) in dependency of SERCA [9]. Additionally, they discovered that ATP in the ER is depleted by oxPhos-inhibitors. Therefore, inhibition of mitochondrial ATP production by oligomycin and increased ER Ca^2+^-depletion by thapsigargin [17] might be key events, generating stress in the ER, which are directly associated with the upregulation of the UPR. This is in accordance with the data of Silva et al. who showed an upregulation of the integrated stress response (ISR), including PERK-signaling (protein kinase R (PKR)-like endoplasmic reticulum kinase), by inhibition of mitochondrial function at oligodendroglia [18]. ATF4 and CHOP are downstream proteins of kinases of the ISR and get triggered by different types of stress. The ISR, triggered by ER stress (e.g. by the accumulation of unfolded proteins) and modulated via PERK-signaling [19], is a known mechanism of cancer cells to promote tumor initiation and progression [20–22].

Tanimura et al. suggested that the UPR is a downstream target of BCR::ABL1 and is important for the survival of Philadelphia (Ph)-positive leukemia cells [23]. We observed a decreased protein expression of UPR-induced transcription factors ATF4 and CHOP when adding imatinib to oligomycin or thapsigargin and hypothesize that this combination of ER stress induction and UPR inhibition of CML cells made them more sensitive to cell death. Similar results were obtained by Kato et al. who tested different ER stress conditions (glucose withdrawal, addition of tunicamycin, and N-linked glycosylation inhibitor) in K562 cells in combination with imatinib [24]. They assumed that BCR::ABL1-inhibition by TKIs induces metabolic vulnerability by blocking the ISR of K562 cells. In particular, they found an imatinib-induced attenuation of eIF2a (eukaryotic translation initiation factor 2a) kinase activation, as well as a reduction of PERK and ATF4 by different TKIs during periods of cellular stress. Additionally, the inactivation of the PERK-elF2a-phosphorylation arm has been shown to sensitize CD34 + BP CML cells to imatinib-induced cell death [25]. Dudka et al. showed that ISRIB (integrated stress response inhibitor), an inhibitor of PERK-downstream signaling, attenuates ISR by reduction of X-box binding protein 1-mediated RAS (rat sarcoma virus)/RAF (rapidly accelerated fibrosarcoma)/MAPK (mitogen-activated protein kinase)-signaling and eIF2a/ATF4-signaling, which is effective in eradicating CML cells in combination with imatinib [26]. This highlights the importance of stress response for the survival of CML cells. Similarly, we hypothesize that imatinib-associated inhibition of UPR upregulation prevented the resolution of ER stress in the CML cell lines, induced by oligomycin and thapsigargin. This might shift the cells in a state of persistent ER stress, triggering downstream proapoptotic signaling pathways. However, ER stress-induced upregulation of the PERK-eIF2a phosphorylation pathway was shown to be associated with imatinib resistance in CML cell lines and CD34^+^ cells from CML patients [25]. Therefore, it needs to be further elucidated in CML patient samples, which TKI is most suitable for a combinatory approach with inducers of ER stress.

We further examined which apoptosis pathways were upregulated by the combinatory addition of imatinib and oligomycin or thapsigargin to CML cells compared to imatinib only. We found a strong increase in the expression of active caspases 3, which are both downstream targets of UPR signaling [17, 27]. Caspase 3 inactivates PARP1 during apoptosis by cleavage between Asp214 and Gly215, which prevents its recruitment to resolve DNA repair [28–31]. In addition, caspase 3/caspase-activated DNase (CAD) has been shown to induce DNA strand breaks [32]. We observed a significant increase in the expression of Cl PARP1 when imatinib was combined with either oligomycin or thapsigargin compared to imatinib alone. Oligomycin and thapsigargin were the major contributors to this effect.

However, both, thapsigargin and oligomycin, have limited potential for therapeutic applications, due to their non-specific cellular toxicity in normal cells [33, 34], also reflected in our experiments by the affection of all cell lines, including the *BCR::ABL1*-negative cell line HL60 by thapsigargin. We therefore tested therapeutically applicable metabolic inhibitors, which might be effective for treating CML in a combinatory approach with TKIs. We combined imatinib, dasatinib, nilotinib, and asciminib with the ATP synthase inhibitor 8-Cl-Ado, which is currently tested in clinical studies in AML patients (e.g. NCT02509546, NCT05263284). We observed an increased lethality and an increased PARP1 cleavage of the CML cell lines K562, BV173, and KU812 in samples with both, TKIs and 8-Cl-Ado. At BV173 and KU812 cells, combinatory apoptosis induction was also observed with the PARP-inhibitor olaparib, a medication, which is approved for treating breast and ovarian cancer by the Food and Drug Administration (FDA) [35]. Although, olaparib could, in contrast to 8-Cl-Ado, barely increase the TKI-induced cell death of K562 cells in our experiments, this agent was completely non-toxic for *BCR::ABL1*-negative HL60 cells.

In contrast to the combination with oligomycin, thapsigargin, and 8-Cl-Ado, there was no increased PARP1 cleavage by olaparib. In contrast, olaparib blocks PARP activity by trapping PARP1 and PARP2 to the DNA to interfere with DNA damage repair and by competing with NAD^+^ to bind at PARP, which is important for PARP to produce PAR chains [36].

## Conclusion

**Fig. 5:**
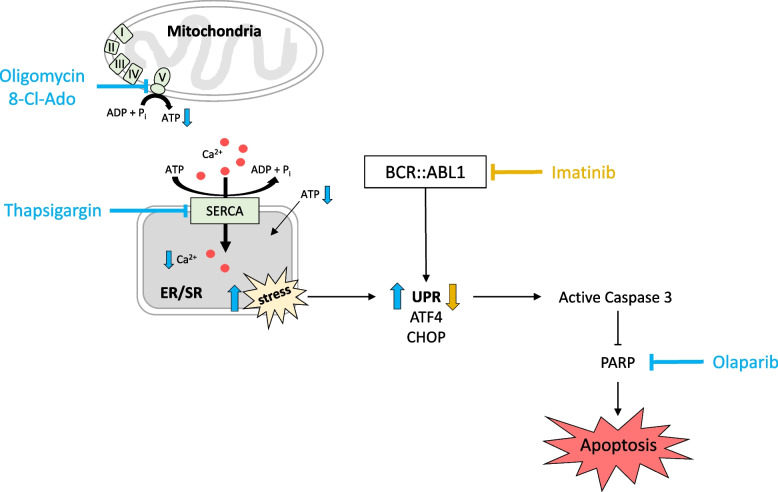
Induction of apoptosis in CML cells by different metabolic inhibitors. Oligomycin and thapsigargin induce ER stress and upregulation of the UPR in different CML cell lines (K562, BV173, and KU812). Imatinib blocks UPR upregulation and the combination of oligomycin or thapsigargin and imatinib leads to a caspase cascade via caspase 3, which results in apoptosis. The ATP synthase inhibitor 8-Cl-Ado and the PARP-inhibitor olaparib are potential therapeutic agents for treating CML, inducing additional apoptosis in combination with TKIs. 8-Cl-Ado = 8-chloroadenosine, ER/SR = endoplasmic/sarcoplasmic reticulum, PARP = poly ADP ribose polymerase, SERCA = sarcoplasmic/endoplasmic reticulum calcium ATPase, UPR = unfolded protein response

We hypothesize that oligomycin and thapsigargin induced ER stress in CML cells, which led to the upregulation of the UPR (Fig. 5). This stress induction was modulated via oligomycin-induced ATP depletion and thapsigargin-dependent Ca^2+^-depletion in the ER. The UPR upregulation was inhibited by imatinib. Compared to CML cells, treated with imatinib only, additional stress induction by oligomycin and thapsigargin triggered apoptosis pathways via the activation of caspase 3 and by inhibition of PARP. In addition, our data suggest that the combinatory usage of TKIs with the ATP synthase inhibitor 8-Cl-Ado or the PARP-inhibitor olaparib are promising therapeutic approaches for future clinical studies on CML patients.

### Supplementary Information

**Additional file 1: Supplementary Table 1.** Inhibitor concentrations. **Supplementary Table 2.** Antibodies and reagents for flow cytometry. **Supplementary Table 3.** Primer for quantitative real‐time PCR. **Supplementary Table 4.** Antibodies for western blot. **Supplementary Figure 1.** Gating strategy for FACS analyses. **Supplementary Figure 2.** TKIs affect CML cell metabolism. **Supplementary Figure 3.** Unpooled data of Figure 1b,c. **Supplementary Figure 4.** Imatinib has no significant effect on oxPhos complex expression. **Supplementary Figure 5.** Thapsigargin increases the lethality of CML cells in combination with TKIs. **Supplementary Figure 6.** Oligomycin induces ER stress and imatinib blocks ATF4 upregulation (uncropped images of Figure 3a). **Supplementary Figure 7.** Oligomycin and thapsigargin induce cleavage of caspase 3 in combination with imatinib in the cell lines K562 and KU812 (uncropped images of Figure 4a).

## Data Availability

Data and materials will be provided upon request by the corresponding author.

## Abbreviations

2-DG: 2-Deoxy-d-glucose
7AAD: 7-Aminoactinomycin
8-Cl-Ado: 8-Chloroadenosine
AA: Antimycin A
AML: Acute myeloid leukemia
ATF: Activating transcription factor
AXER: ATP/ADP exchanger in the endoplasmic reticulum membrane
BCA: Bicinchoninic acid
BP: Blast phase
CD: Cluster of differentiation
C/EBP: CCAAT-enhancer-binding protein
CHOP: C/EBP homologous protein
Cl: Cleaved
CML: Chronic myeloid leukemia
ECAR: Extracellular acidification rate
eIF2a: Eukaryotic translation initiation factor 2a
ER: Endoplasmic reticulum
FA: Fatty acid
FACS: Fluorescence-activated cell sorting
FCS: Fetal calf serum
FDA: Food and Drug Administration
Glut: Glucose transporter
GlycoATP: Glycolytic ATP production rate
HRP: Horseradish peroxidase
IRE1α: Inositol-requiring enzyme 1α
ISR: Integrated stress response
ISRIB: Integrated stress response inhibitor
LSC: Leukemia stem cell
MAPK: Mitogen-activated protein kinase
MitoATP: Mitochondrial ATP production rate
OCR: Oxygen consumption rate
OxPhos: Oxidative phosphorylation
PARP: Poly ADP ribose polymerase
PBS: Phosphate buffered saline
PERK: Protein kinase R (PKR)-like endoplasmic reticulum kinase
Ph: Philadelphia
P/S: Penicillin/Streptomycin
PVDF: Polyvinylidene fluoride
qPCR: Quantitative polymerase chain reaction
RAF: Rapidly accelerated fibrosarcoma
RAS: Rat sarcoma virus
RIPA: Radioimmunoprecipitation assay
ROS: Reactive oxygen species
Rot: Rotenone
ROUT: Robust regression and outlier removal
SCD: Specific cell death
SD: Standard deviation
SDS-PAGE: Sodium dodecyl sulfate polyacrylamide gel electrophoresis
SERCA: Sarcoplasmic/endoplasmic reticulum calcium ATPase
SLC35B1: Solute carrier family 35 member B1
SR: Sarcoplasmic reticulum
TFR: Treatment-free remission
TKI: Tyrosine kinase inhibitor
TMRE: Tetramethylrhodamine, ethyl ester
UPR: Unfolded protein response
