# Supplementary material for: Synergistic lethality in chronic myeloid leukemia – targeting oxidative phosphorylation and unfolded protein response effectively complements tyrosine kinase inhibitor treatment
Source: BMC Cancer. 2023 Nov 27;23:1153. doi: 10.1186/s12885-023-11623-6 (PMC10680331; doi:10.1186/s12885-023-11623-6)
Supplement: Supplementary file 1 — Additional file 1: Supplementary Table 1. Inhibitor concentrations. Supplementary Table 2. Antibodies and reagents for flow cytometry. Supplementary Table 3. Primer for quantitative real‐time PCR. Supplementary Table 4. Antibodies for western blot. Supplementary Figure 1. Gating strategy for FACS analyses. Supplementary Figure 2. TKIs affect CML cell metabolism. Supplementary Figure 3. Unpooled data of Figure 1b,c. Supplementary Figure 4. Imatinib has no significant effect on oxPhos complex expression. Supplementary Figure 5. Thapsigargin increases the lethality of CML cells in combination with TKIs. Supplementary Figure 6. Oligomycin induces ER stress and imatinib blocks ATF4 upregulation (uncropped images of Figure 3a). Supplementary Figure 7. Oligomycin and thapsigargin induce cleavage of caspase 3 in combination with imatinib in the cell lines K562 and KU812 (uncropped images of Figure 4a). [file 12885_2023_11623_MOESM1_ESM.pdf]

Supplementary information to:

Häselbarth et al. - *BMC Cancer* (2023)

Supplementary Table 1: Inhibitor concentrations.

Inhibitor	Concentration [µM]				Manufacturer
	K562	BV173	KU812	HL60	
8-Cl-Ado	10	10	10	10	Tocris
Antimycin A (ATP Rate)	0.5	0.5	0.5	0.5	Sigma-Aldrich
Antimycin A (SCD)	1	1	1	1	Sigma-Aldrich
Asciminib	0.01	0.1	0.01	0.1	MedChemExpress
Bz423	20	20	20	20	Tocris
Dasatinib	0.00075	0.0075	0.000375	0.0075	Selleckchem
Imatinib	0.2	0.2	0.1	0.2	Sigma-Aldrich
NaN_3	1000	1000	1000	1000	Sigma-Aldrich
Nilotinib	0.01	0.01	0.01	0.01	StemCell
Olaparib	5	0.5	5	5	Selleckchem
Oligomycin	1	1	1	1	Sigma-Aldrich
Oligomycin (ATP Rate)	1.5	1.5	1.5	1.5	Sigma-Aldrich
Rotenone (ATP Rate)	0.5	0.5	0.5	0.5	Sigma-Aldrich
Rotenone (SCD)	1	1	1	1	Sigma-Aldrich
Thapsigargin	0.05	0.1	0.05	0.1	Cayman

Supplementary Table 2: Antibodies and reagents for flow cytometry.

Antibody	Fluorochrome	Clone	Isotype	Manufacturer
6-NBDG				ThermoFisher Scientific
7AAD				Biolegend
αCaspase 3 (Cl Asp175)	AlexaFluor647	D3E9	Rabbit IgG	Cell Signaling Technology
αCD36	FITC	5-271	Mouse IgG2a,κ	Biolegend
αCD98	PE/Vio770	REA387	Human IgG1	Miltenyi Biotec
αGlut-1	APC	202915	Mouse IgG_{2B}	RnD Systems
αPARP1 (Cl Asp214)	PE	HLNC4	Mouse IgG1,κ	ThermoFisher Scientific
Bodipy^{TM} FL C_{16}				ThermoFisher Scientific
CellROX^{TM} Deep Red				ThermoFisher Scientific
MitoSOX™ Red Mitochondrial Superoxide Indicator				ThermoFisher Scientific
MitoTracker^{TM} Green FM				ThermoFisher Scientific
TMRE				ThermoFisher Scientific
Zombi Aqua^{TM}				Biolegend

Supplementary Table 3: Primer for quantitative real-time PCR.

Gene	QuantiTect Primer Assay	Qiagen Product Nr.
acly	Hs_ACLY_1_SG	QT00062286
beta-actin	Hs_ACTB_2_SG	QT01680476
atpif1	Hs_ATPIF1_va.1_SG	QT01028993
cpt1a	Hs_CPT1A_1_SG	QT00082236
eno1	Hs_ENO1_1_SG	QT00090881
fasn	Hs_FASN_1_SG	QT00014588
g6pd	Hs_G6PD_1_SG	QT00071596
gls	Hs_GLS_1_SG	QT00019397
hk2	Hs_HK2_1_SG	QT00013209
ldha	Hs_LDHA_1_SG	QT00001687
pdha1	Hs_PDHA1_1_SG	QT00087780
pdk1	Hs_PDK1_1_SG	QT00069636
slc2a1	Hs_SLC2A1_1_SG	QT00068957
slc38a1	Hs_SLC38A1_1_SG	QT00093142
slc38a2	Hs_SLC38A2_1_SG	QT00030499
tfam	Hs_TFAM_1_SG	QT00012782

Supplementary Table 4: Antibodies for western blot.

Antibody	Clone	Isotype	Dilution	Manufacturer
**Primary**				
ATF4	D4B8	Rabbit IgG	1:1000	Cell Signaling Technology
ATF4	E4Q4E	Mouse IgG1,κ	1:1000	Cell Signaling Technology
ATP5A	15H4C4	IgG2b	1:1000	Abcam
Beta-actin	D6A8	Rabbit IgG	1:1000	Cell Signaling Technology
CHOP	L63F7	Mouse IgG2a	1:1000	Cell Signaling Technology
COX II	12C4F12	IgG2a	1:1000	Abcam
GAPDH	D16H11	Rabbit IgG	1:1000	Cell Signaling Technology
NDUFB8	20E9DH10C12	IgG1	1:1000	Abcam
SDHB	21A11AE7	IgG2a	1:1000	Abcam
UQCRC2	13G12AF12BB11	IgG1	1:1000	Abcam
**Secondary**				
Goat anti-mouse HRP			1:1000	Agilent
Goat anti-rabbit IgG HRP			1:1000	Cell Signaling Technology

Gating for FACS analyses

a) Cell death analysis

uc (K562):

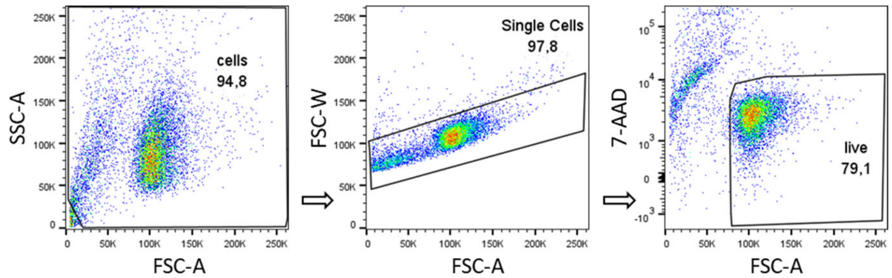

Olaparib + Asciminib (K562):

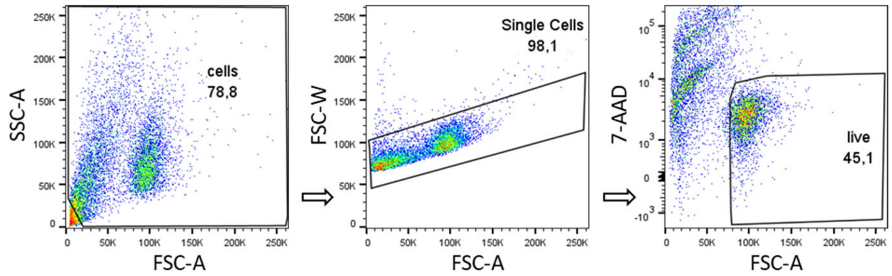

b) PARP1 cleavage

uc (BV173):

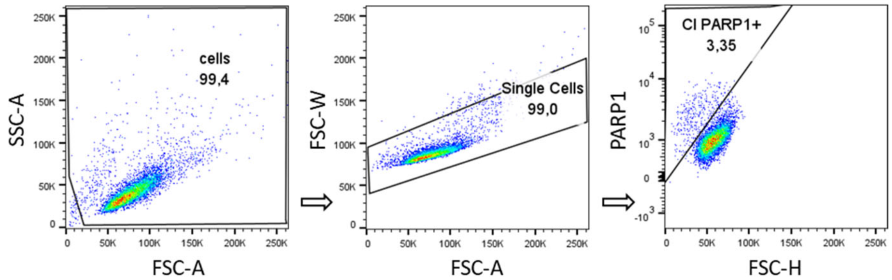

8-Cl-Ado + Imatinib (BV173):

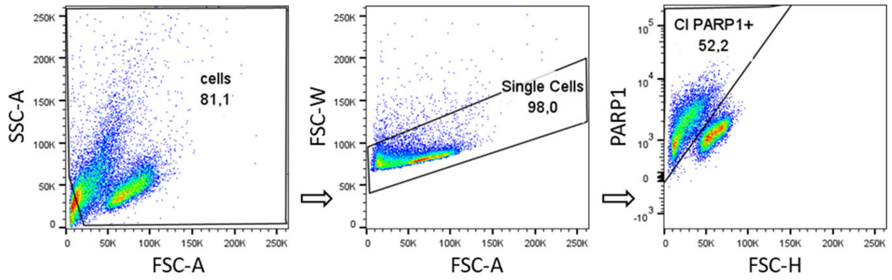

Supplementary Figure 1: Gating strategy for FACS analyses. (a) For cell death measurements, debris was excluded, then cells were further gated for single, live (7AAD-negative) cells. The dot blots exemplarily show K562 cells, which were treated for 24h with olaparib + asciminib or remained untreated. (b) For the analysis of PARP1, debris was excluded, then cells were further gated for single, PARP1^{+} cells. The dot blots exemplarily show BV173 cells, which were treated for 24h with 8-Cl-Ado + imatinib or remained untreated. 8-Cl-Ado = 8-chloroadenosine, Cl = cleaved, PARP = poly ADP ribose polymerase, uc = untreated control.

Metabolic parameters

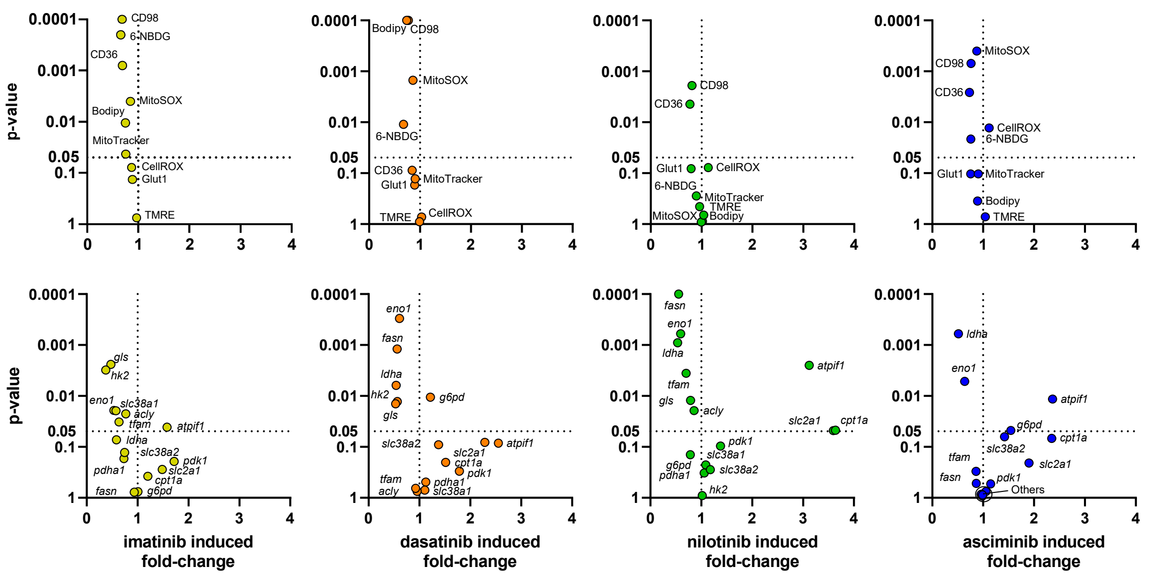

	Imatinib			Dasatinib			Nilotinib			Asciminib		
	fold-change	SD	p-value	fold-change	SD	p-value	fold-change	SD	p-value	fold-change	SD	p-value
**6-NBDG**	0.66	0.15	0.0002	0.67	0.30	0.0110	0.90	0.27	0.2805	0.76	0.25	0.0214
**Bodipy**	0.75	0.22	0.0105	0.74	0.11	0.0001	1.02	0.36	0.8828	0.89	0.32	0.3523
**CD36**	0.69	0.08	0.0008	0.85	0.22	0.0884	0.77	0.17	0.0044	0.73	0.19	0.0026
**CD98**	0.69	0.11	0.0001	0.77	0.09	0.0001	1.00	0.11	0.0019	0.76	0.13	0.0007
**CellROX**	0.87	0.19	0.0779	1.03	0.28	0.7277	1.13	0.19	0.0779	1.12	0.11	0.0129
**Glut1**	1.12	0.17	0.1347	0.90	0.21	0.1716	0.80	0.31	0.0813	0.90	0.13	0.1037
**MitoSOX**	0.85	0.11	0.0040	0.86	0.09	0.0015	1.00	0.13	0.9125	0.87	0.07	0.0004
**MitoTracker**	0.76	0.28	0.0429	0.91	0.17	0.1290	0.96	0.15	0.4545	0.90	0.16	0.1037
**TMRE**	0.97	0.29	0.7552	0.99	0.19	0.8990	1.04	0.27	0.6682	1.04	0.34	0.7224

acly	0.77	0.23	0.0225	0.96	0.32	0.0225	0.86	0.15	0.0194	0.99	0.25	0.8801
atpif1	1.58	0.82	0.0412	2.55	2.18	0.0412	3.12	1.47	0.0025	2.36	1.14	0.0115
cpt1a	1.20	0.51	0.3781	1.52	0.76	0.3781	3.64	3.38	0.0472	2.35	1.92	0.0682
eno1	0.54	0.39	0.0193	0.61	0.08	0.0193	0.60	0.22	0.0006	0.64	0.29	0.0052
fasn	0.93	0.64	0.7798	0.56	0.16	0.7798	0.56	0.19	0.0001	0.87	0.60	0.5212
g6pd	1.01	0.42	0.7630	1.21	0.13	0.7630	1.09	0.19	0.2259	1.55	0.70	0.0477
gls	0.37	0.35	0.0031	0.53	0.25	0.0031	0.79	0.20	0.0123	0.98	0.51	0.9317
hk2	0.48	0.28	0.0024	0.57	0.28	0.0024	1.02	0.43	0.9080	0.96	0.63	0.8613
ldha	0.59	0.39	0.0729	0.54	0.25	0.0729	0.54	0.27	0.0009	0.51	0.26	0.0006
pdha1	0.73	0.36	0.1699	1.13	0.41	0.1699	1.06	0.17	0.3267	1.05	0.50	0.7563
pdk1	1.72	1.36	0.1948	1.79	1.67	0.1948	1.38	0.60	0.0967	1.15	0.69	0.5348
slc2a1	1.48	1.19	0.2776	2.29	1.45	0.2776	3.60	3.34	0.0480	1.90	3.02	0.2078
slc38a1	0.58	0.36	0.0195	1.11	0.66	0.0195	0.78	0.40	0.1434	0.96	0.55	0.8525
slc38a2	0.74	0.53	0.1295	1.38	0.44	0.1295	1.18	0.46	0.2810	1.42	0.59	0.0632
tfam	0.63	0.39	0.0324	0.92	0.39	0.0324	0.70	0.22	0.0036	0.86	0.38	0.3023

Supplementary Figure 2: TKIs affect CML cell metabolism. The human CML cell lines K562, BV173, and KU812 were treated with/without the TKIs imatinib (yellow), dasatinib (orange), nilotinib (green), or asciminib (blue) for 24h. Metabolic markers were assessed by FACS (at the top) or qPCR (below) and normalized to the uc. Data from all CML cell lines were pooled and are represented by the p-value plotted against the mean of the TKI-induced fold-change. The table below provides an overview of all means, SDs, and p-values. TKI-treated samples were each compared with the uc (n=6-9). SD = standard deviation, TKI = tyrosine kinase inhibitor, uc = untreated control.

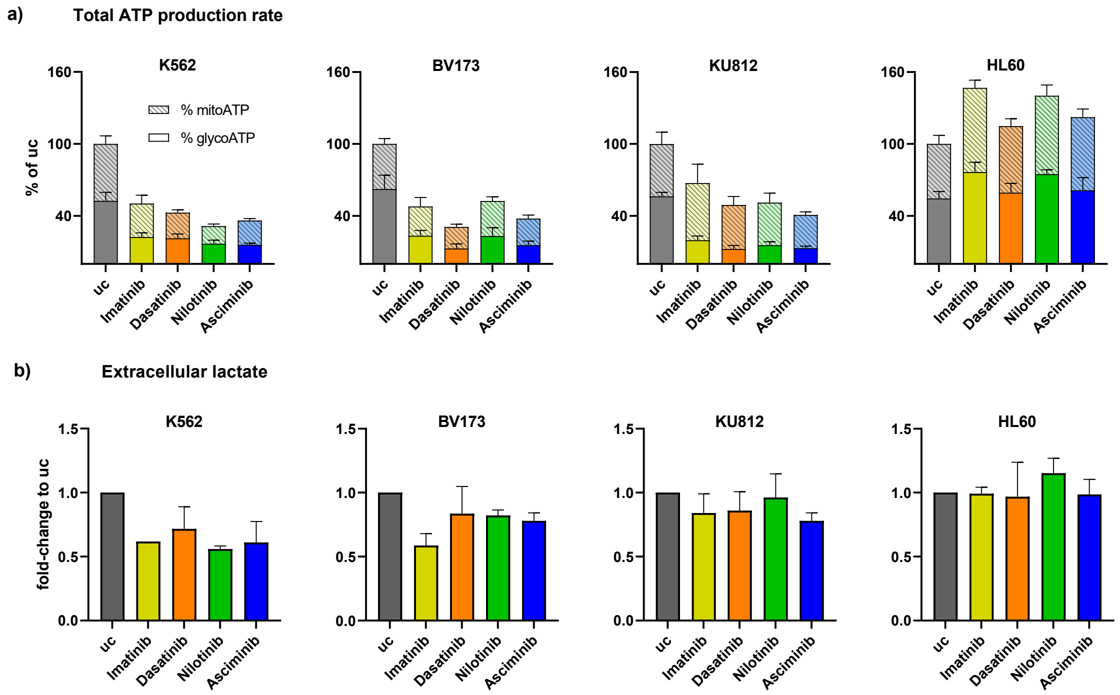

Supplementary Figure 3: Unpooled data of Figure 1b,c. The human CML cell lines K562, BV173, and KU812 and the *BCR::ABL1*-negative control cell line HL60 were treated with/without the TKIs imatinib (yellow), dasatinib (orange), nilotinib (green), or asciminib (blue) for 24h. (a) ATP production rate was observed by Real-Time ATP Rate Assay. Data are presented by the mean and SD of the technical replicates (b) Lactate content of cell supernatants was measured with the Super GL compact. Data are represented by the mean and SD of the biological replicates. glycoATP = glycolytic ATP production rate, mitoATP = mitochondrial ATP production rate, SD = standard deviation, TKI = tyrosine kinase inhibitor, uc = untreated control.

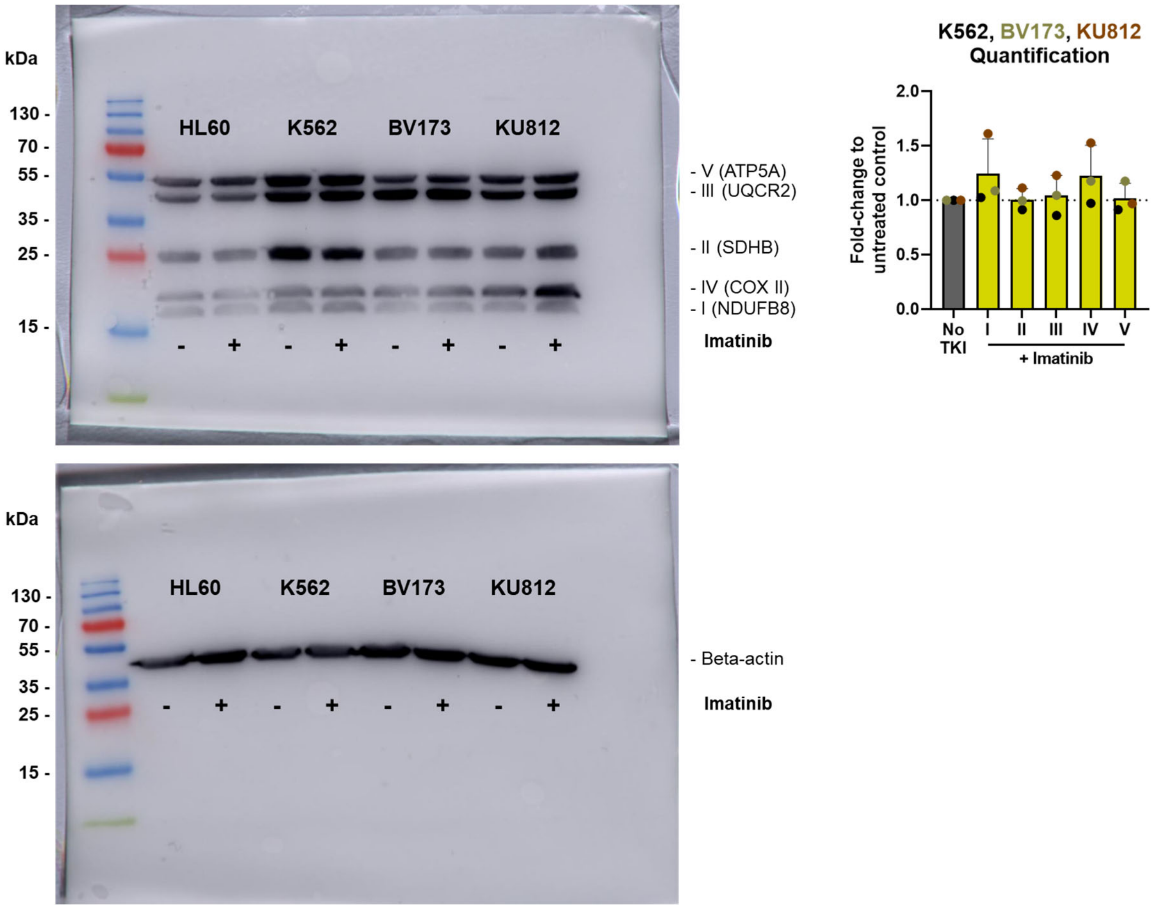

Supplementary Figure 4: Imatinib has no significant effect on oxPhos complex expression. The human CML cell lines K562, BV173, and KU812, and the *BCR::ABL1*-negative HL60 control cell line were treated with/without imatinib for 24h and expression of oxPhos complexes (I-V) was assessed by western blot with beta-actin as a reference protein. The samples from the top blot were derived from the same experiment as the bottom blot and both blots were processed in parallel. Quantification (top right) is shown for K562, BV173, and KU812 cells and compares the controls (no TKI) with the imatinib-treated samples each (n=3). Quantifications are represented by the mean gray value and the standard deviation. kDa = kilo dalton, TKI = tyrosine kinase inhibitor.

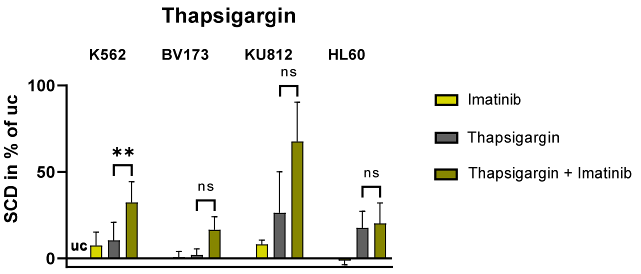

Supplementary Figure 5: Thapsigargin increases the lethality of CML cells in combination with TKIs. The human CML cell lines K562, BV173, and KU812 and the *BCR::ABL1*-negative control cell line HL60 were treated with imatinib for 24h in combination with the SERCA-inhibitor thapsigargin. The cells were stained with 7AAD and cell death was measured via FACS. Data are shown in % of the uc (= 0%). Data are represented by the mean and standard deviation. Comparison of thapsigargin + imatinib with thapsigargin only (n=3-4 per cell line). SCD = specific cell death, SERCA = Sarcoplasmic/endoplasmic reticulum calcium ATPase, TKI = tyrosine kinase inhibitor, uc = untreated control. ** $p < 0.01$, ns = not significant.

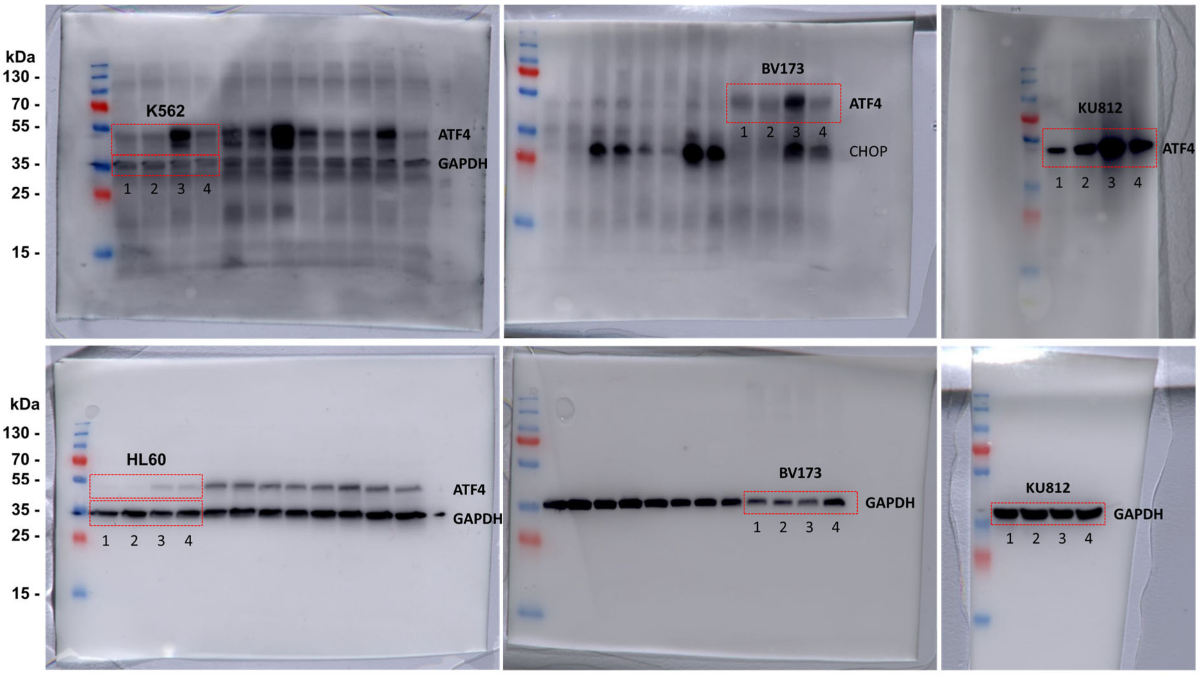

1: uc, 2: Imatinib, 3: Oligomycin, 4: Oligomycin + Imatinib

Supplementary Figure 6: Oligomycin induces ER stress and imatinib blocks ATF4 upregulation (uncropped images of Figure 3a). Figure representatively shows the ATF4 expression (49 kDa) of the human CML cell lines K562, BV173, and KU812 and the *BCR::ABL1*-negative control cell line HL60, treated with/without imatinib and oligomycin for 24h with GAPDH (35 kDa) as a reference protein. When ATF4 and GAPDH were detected on different blots (see here for BV173 and KU812), both blots were derived from the same experiment and were processed in parallel. The red rectangles indicate where the images for Figure 3a were cropped. kDa = kilo dalton, uc = untreated control.

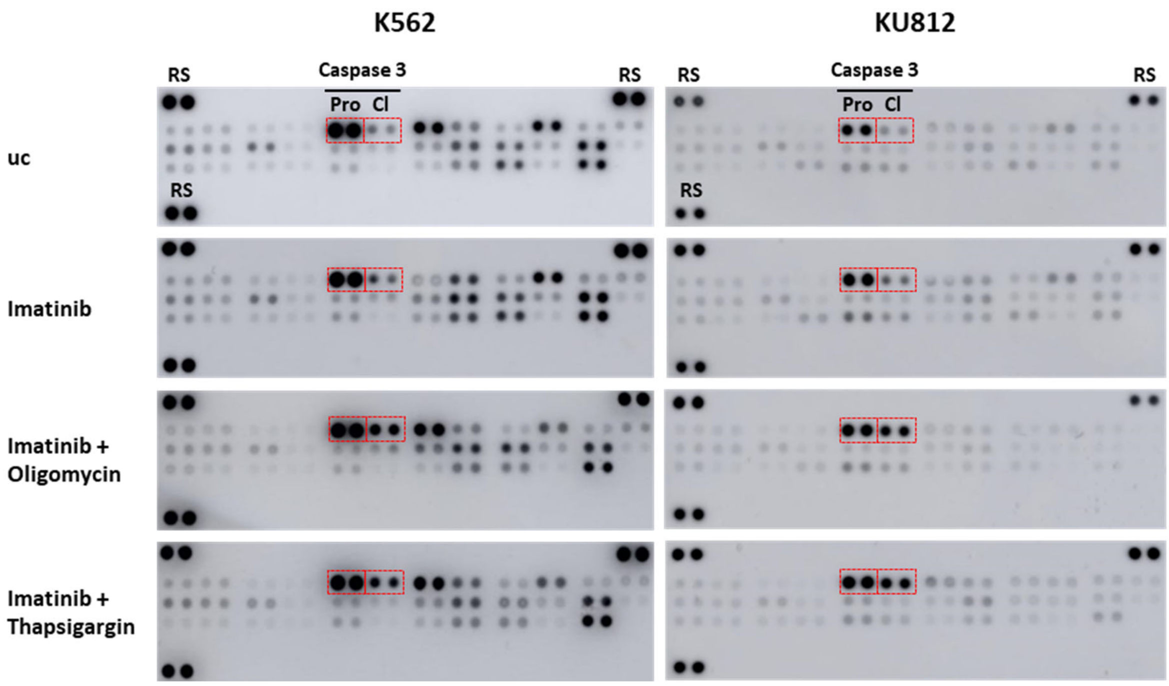

Supplementary Figure 7: Oligomycin and thapsigargin induce cleavage of caspase 3 in combination with imatinib in the cell lines K562 and KU812 (uncropped images of Figure 4a). The human CML cell lines K562 (left) and KU812 (right) were treated with/without imatinib and oligomycin or thapsigargin for 24h. Subsequently, the expression of apoptosis-related proteins was determined via a membrane-based sandwich immunoassay. The red rectangles indicate where the images for Figure 4a were cropped. Cl = cleaved, RS = reference spots, uc = untreated control.
